# The Evaluation of the Biological Effects of Melanin by Using Silkworm as a Model Animal

**DOI:** 10.3390/toxins14070421

**Published:** 2022-06-21

**Authors:** Vivian Andoh, Liang Chen, Feifei Zhu, Qi Ge, Lin Ma, Qiang Wang, Keping Chen

**Affiliations:** 1 School of Food and Biological Engineering, Jiangsu University, Zhenjiang 212013, China; vivianandoh@gmail.com; 2 School of Life Sciences, Jiangsu University, Zhenjiang 212013, China; oochen@ujs.edu (L.C.); feizhu@ujs.edu.cn (F.Z.); geqi0616@163.com (Q.G.); 3 School of the Environment and Safety Engineering, Jiangsu University, Zhenjiang 212013, China; 4 College of Biotechnology, Jiangsu University of Science and Technology, Zhenjiang 212100, China

**Keywords:** melanin, biological effects, silkworm, silk properties

## Abstract

Melanin has been reported to have potential applications in industries such as cosmetics and food due to its anti-UV and antioxidative qualities. However, the corresponding data on its safety evaluation or biological consequences are fairly limited; such data are critical given its widespread use. The effect of different concentrations (1, 2, 3, and 4%) of melanin on growth status (larvae length and weight, cocoon weight, and morphology), the microstructure of the various tissues (fat body, silk gland, and midgut), and silk properties was studied by using the silkworm (*bombyx mori*) as the model organism. The weight and length of silkworm larvae fed with melanin were lower than the control, indicating that melanin appears to have a negative effect on the growth status of silkworms; however, the histophysiology analysis indicates that the cell morphologies are not changed, the XRD and FTIR spectra indicate that the secondary and crystalline structures of silks are also well preserved, and the thermogravimetric analysis and tensile test indicate that the thermal stability and mechanical properties are well maintained and even improved to some extent. Generally, it indicates that melanin has a certain inhibitory effect on the growth of silkworm larva but causes no harm to the cell microstructures or silk properties; this demonstrates that the safety of melanin as a food addictive should be considered seriously. The increase of thermal stability and mechanical properties shows that melanin may be a good chemical modifier in textile industries.

## 1. Introduction

Melanin is a natural macromolecule pigment which is commonly distributed in animals, plants, and microbial organisms [1,2,3,4], providing a variety of functions such as thermal regulation, mechanical protection, pigmentation, photosensitivity, radiation protection, antioxidant activity, and so on [5,6,7]. It has been reported to have potential applications in industries such as cosmetics, food additives, pharmacology, paints, and so on [8,9], thus gaining much interest. Melanogenesis, or the production of melanin, occurs in melanocytes, which are specialized pigment-producing cells that produce melanin as pigments granules. The primary goal of this process is thought to be to protect keratinocyte DNA from damage caused by UV radiation and certain toxins such as free oxygen radicals [10]. These pigments play critical roles in UV protection and other stress factors. Although melanin is commonly thought to be a very stable organic compound, studies have shown that it has significant chemical activity and can undergo physical and chemical changes even in vivo [11]. Moreso, as more melanin is introduced into people’s lives, their contact with it becomes more intimate, emphasizing the importance of conducting an objective evaluation of its biological impacts and toxicity. However, there is a scarcity of evidence on its safety evaluation and biological consequences, and such evidence is crucial given its extensive use. Understanding the structure and biological mechanisms that result in the several melanins present in nature inspires new synthetic approaches and materials.

Natural melanin can be separated from a variety of plants, animals, and microbes, while artificial melanin-like derivatives can be made by simple polymerization [12,13]. Various biomedical applications and food packaging have been made either alone or by mixing melanin or melanin nanoparticles [14]. For example, Ye et al. [15] showed that melanin from *Lachnum* YM404 had predominant resistance activity to UV radiation, indicating that it could be utilized as a new type of natural anti-UV additive applied in cosmetics and sunscreen. Hung et al. [16] also reported that melanin derived from tea had antioxidant activities and can protect the liver against hydrazine-induced oxidative toxicity. In addition, a group of researchers recently demonstrated that a melanin nanocomposite loaded with epigallocatechin-3-gallate retained high antibacterial and antioxidant properties, implying that it could be used as food-active packaging fillers [17]. Although melanin exists naturally in many animals and plants, it cannot be concluded directly that it will have no negative impacts when used as additives. Detailed data about the evaluation of the biological effects of melanin by using a suitable animal model are very important; however, the relative reports are very scarce.

The silkworm (*Bombyx mori*), a lepidopteran insect, is a typical invertebrate animal model [18,19,20], which, in recent years, has gained the favor of researchers in several fields such as medicine, toxicity evaluation of nanomaterials, environmental monitoring, and so on [21,22,23,24], owing to its peculiar advantages [25,26]. Similar to other lower invertebrate animal models such as *Salamandra Laurenti*, *Caenorhabditis elegans*, or *Drosophila*, silkworms will not be trapped in the ethical disputes that usually accompany mammal models [27,28]. In addition, the silkworm has its own advantages; for example, it will not cause biosafety problems since it cannot survive outside the laboratory, and the size of the silkworm is also more suitable for dissection and handling [29,30]. Due to the excellent characteristics of the silkworm model, many researchers chose the silkworm model to study the biological effects of their targets such as nanomaterials, drugs, cosmic rays, etc. [31,32]. For instance, Shi et al. [33] used the silkworm model to investigate the biological effects of cosmic rays. Moreover, Matsumoto and Sekimizu demonstrated that silkworm infection models could be ideal for uncovering the mechanism of fungi infection [34]. In previous reports, our group studied the biological effects of BSA-capped gold nanoclusters, graphene quantum dots, and boron nitride nanosheets by using silkworm as a model [35,36,37].

In this work, the biological effects of melanin were assessed by a silkworm model, via illustrating the growth status, cell morphology, and silk properties. We demonstrated that melanin could inhibit the growth of silkworm larvae, while the cell morphologies of some tissues (silk gland, fat body, and midgut) show no abnormal changes. The XRD and FTIR results show that the crystalline and secondary structures of silks are not damaged. The TGA analysis and tensile test demonstrate that the thermal stability and mechanical properties of silks are also preserved well, thus giving more data on the safe use of melanin.

## 2. Results and Discussions

### 2.1. The Effect of Melanin on the Growth, Cocoon, and Silk Morphology of Silkworms

The silkworms (*Bombyx mori*) used in this study were divided into control and experimental groups. The control group contained 20 silkworm larvae and was fed with mulberry leaves sprayed with distilled water from the 1st day of fifth instar until cocooning. In the same way, the experimental groups, composed of four groups (with 20 silkworm larvae in each), were fed with mulberry leaves sprayed with melanin at different mass concentrations (G1, 1%; G2, 2%; G3, 3%; and G4, 4% (G means group)). 

**Figure 1:**
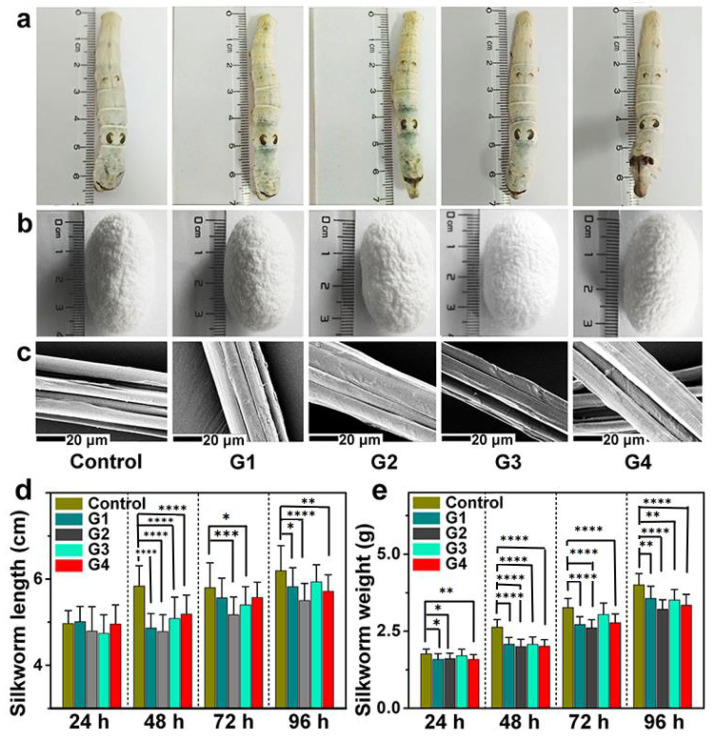
The impact of melanin on the growth, cocoons, and degummed silks of silkworms. (**a**) The influence of melanin on the exterior of silkworms after the feeding of melanin for 96 h. (**b**,**c**) The influence of melanin on the appearance of cocoons (**b**) and degummed silks (**c**). (**d**,**e**) The influence of melanin on the average length (**d**) and weight (**e**) of silkworms after the feeding of melanin at different times. The error bars are presented in mean ± SD; * *p* < 0.05, ** *p* < 0.01, *** *p* < 0.001, and **** *p* < 0.0001.

In order to determine the effects of melanin on the silkworms, the appearance, length, and weight of silkworm larvae were observed every 24 h (Figure 1 and Supplementary Table S1). Figure 1a depicts the appearance of silkworm larvae. It was observed that silkworm larvae from various groups were similar in appearance and color, indicating that the majority of melanin (black-colored) taken was excreted. The effect of melanin on the length and weight of silkworm larvae was also studied. As shown in Figure 1d,e, the average length and weight from all the melanin groups were lower than the control group, indicating that melanin had some negative impacts on the growth of silkworms. 

The influence of melanin on cocoons was also studied. From Figure 1b, it is observed that the cocoons do not show any appreciable physical change when comparing the melanin groups with the control group. The average cocoon weight from each group was also observed, and the results (Supplementary Figure S1a and Table S2) show that the average cocoon-weight data of all the groups are similar; however, an irregular change is found. When the melanin groups were compared to control groups, the cocoons showed no discernible physical change, as shown in Figure 1b. The average cocoon weight from each group was also measured; the results showed that, despite an irregular change, the average cocoon-weight data from all groups were similar (Supplementary Figure S1a and Table S2). The morphology and diameter of the degummed silk fibers from either the control or melanin groups were studied by using scanning electron microscopy (SEM). Silks from all five groups (Figure 1c) exhibited identical morphology and average diameter (Supplementary Figure S1b and Table S2), indicating that melanin had no effect on the usual microstructure of silkworm silk. This may be attributable to the fact that the melanin consumed by the silkworm was transferred to the silk gland and interacted with the silk fibroin to impact the spinning process [35], resulting in the minor decrease in the average diameter of the silk. 

The death rate of each group was also recorded, and there was no record of death throughout the entire period of the experiment, indicating that the melanin in the concentration range employed had no lethal effect on silkworms. This demonstrated that the modified diet employed in our work was safe for silkworms. It is well established that melanin is not required for growth and development, but it does play a significant function in survival and defense [38]. Using AMD-like cellular and mouse models, Kwon et al. [39] demonstrated that melanin or melanin nanoparticles are biocompatible, nontoxic, and preferentially target reactive oxygen species with powerful antioxidant properties.

In summary, although it was believed that melanin was primarily excreted from the silkworm body, it caused certain damage to the growth of silkworm larvae, despite the fact that the morphologies of the silkworm larvae, cocoons, and silks were all normal.

### 2.2. Histophysiological Study

**Figure 2:**
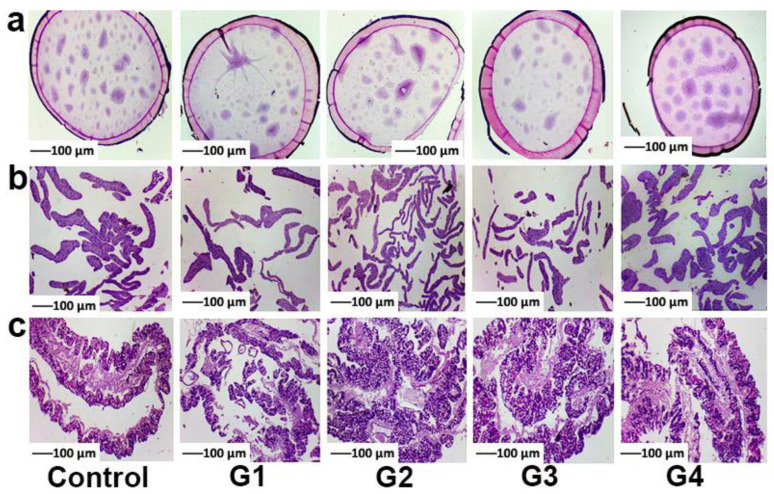
Histophysiological descriptions of silk gland (**a**), fat body (**b**), and midgut (**c**) after the feeding of melanin for 96 h.

A histophysiological study was carried out to analyze the influence of melanin on the cell morphology of several tissues (silk gland, fat body, and midgut); histopathological sections were examined 96 h after feeding melanin to the silkworm, and the results are displayed in Figure 2. When compared to the control group’s images, it was clear that melanin had no significant negative impact on the pathological microstructures of silkworm tissues (silk gland, fat body, and midgut); that is, the morphologies of cells from the various tissues were similar across all groups. Comparing the control and melanin groups, we observed that the silk glands exhibited a standard architecture, with a full lumen entirely filled with proteins, which are thought to promote silk production, and thin walls (Figure 2a); the cells of the fat body (Figure 2b) were well ordered, and the distance between them was uniform; and the midgut (Figure 2c) from both the control and melanin groups showed no uncharacteristic pathological changes with clearly visible basal laminae, proving that no damages was caused to them by melanin.

### 2.3. The Effect of Melanin on the Structures of Silk

#### 2.3.1. XRD Analysis

**Figure 3:**
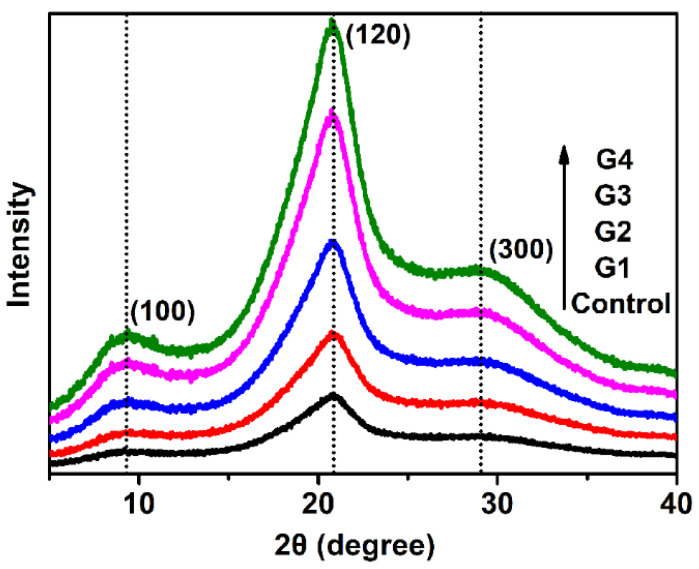
XRD patterns of the control and melanin-modified silk (G1–G4) fibers at different temperatures.

To investigate the effect of melanin on the crystalline structure of silks, the X-ray diffraction (XRD) spectra of silks from both the melanin and control groups were examined (Figure 3). All silk samples were scanned by using a two range of 5–40°. As shown, the strongest peaks in silk samples from all five groups were at (100), (120), and (300), which correlate to β-sheet in silk and also contribute to Young’s modulus and the strength of silk fibers [35,36,37,40]. Despite the presence of melanin in each group, there was no significant difference in the resulting patterns, demonstrating that melanin feeding had no effect on the core crystalline structures of silks.

#### 2.3.2. FTIR Analysis

**Figure 4:**
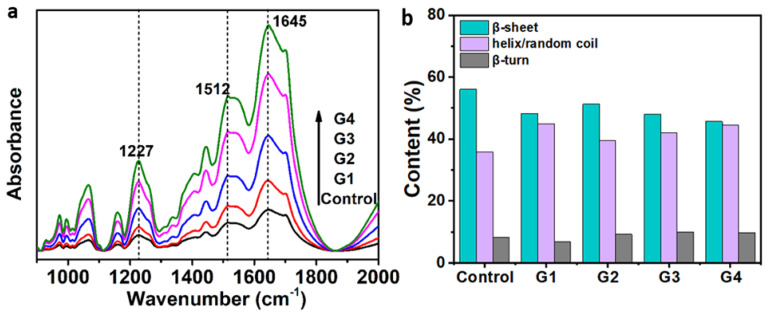
Effect of melanin on the secondary structure of silks. (**a**) FTIR spectra of various silks samples. (**b**) The secondary structures contents of silk samples obtained to the deconvoluted amide I band spectra.

A silk protein conformation is essential to determine the mechanical performance of materials. Here, FTIR was used to identify silk proteins and evaluate their relative content by revealing the effects of melanin on their secondary structures. The peaks at 1630, 1665, and 1700 cm^−1^ were attributed to random coil, β-sheet, and β-turn [41,42,43,44]. The FTIR spectra shown in Figure 4a showed that all the spectra corresponding to control and melanin silks have the same peaks. Three significant peaks located at 1227 cm^−1^ (amide III, random coil/helix, or both), 1512 cm^−1^ (amide II and β-sheet), and 1645 cm^−1^ (amide I, random coil/helix, or both) were found, showing the coexistence of β-sheet, helix, and random coil conformations in silks. No obvious difference was found among the spectra from the five silk samples, proving that the presence of melanin in the silk fibers did not cause any damage or changes to the secondary structures of silks. However, the fact that melanin had a greater impact on silk behavior than protein structure was reinforced. 

The amide I spectral region was deconvoluted to determine the contents of the secondary structures. This is due to the fact that the amide I area of the spectrum is the most informative for determining protein secondary structure [45]. The FTIR spectra of amide I are presented in detail as deconvoluted FTIR spectra (Supplementary Figure S2), and the contents of the secondary structures are shown in Figure 4b, revealing that the melanin-modified silks contained more helix and random coil structures and a fewer β-sheets than the control silk, similar to previously reported results [46]. This may be attributed to the formation of hydrogen bonds between melanin and silk fibroins. It can also be noticed that there was an increase in helix and random coil content when the control was compared with the melanin groups; and this may lead to the increase in their toughness modulus. A random coil structure can be transformed into a β-sheet structure, making the silk fiber become rough and rigid; this is consistent with previous conclusions [47]. An increasing trend of the β-sheets content can be seen in the melanin silks (G1–G3), confirming a tougher breaking strength. This was also confirmed in the dose–response relationship between β-sheets content and melanin dose. Details on the relationship between melanin dose and the relative β-sheets content are provided in Supplementary Figure S3.

### 2.4. The Effect of Melanin on the Properties of Silks

#### 2.4.1. Thermal Stability

**Figure 5:**
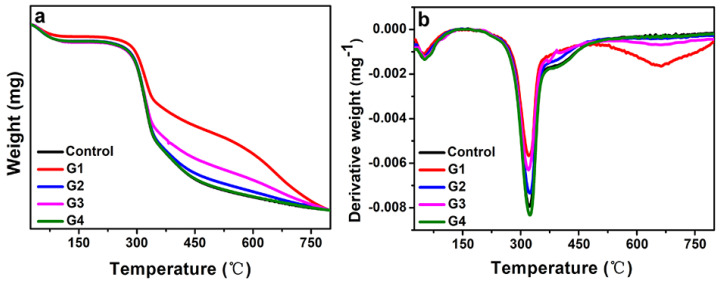
(**a**) Thermogravimetric analysis (TGA) and (**b**) derivative thermogravimetric analysis (DTG) curves of silks from the various groups.

Figure 5a,b shows the thermal stability of different silk fibers characterized by thermogravimetric (TGA) and derivative thermogravimetric (DTG) analysis, respectively. Figure 5a shows that, as the temperature increased, the weight of all the silk samples gradually decreased, with the onset decomposition temperature commencing at 250 °C and the decreasing rate rapidly increasing. Weight loss is probably caused by the elimination of adsorbed water, as previously reported [48]. The residue masses of the treated silk fibers were also higher than those of the control, and this can be attributed to the presence of melanin in the silk fibers. From Figure 5b, it was observed that the endothermic transition of all samples was around 330 °C, which was caused by the thermal decomposition of the silks. Although the changing trend of each silk sample was similar, it was found that the decreasing rate of melanin-modified silks was lower than the one of the control silks, demonstrating that the stability of silks was improved by the intake of melanin, which demonstrated higher efficiency for enhancing the thermal stability of silk fibers. The reason may be the strong hydrogen bonding interaction between the melanin and the silk fibroin. This suggests that melanin by its scavenging action can stabilize silk fibers such that it retains its molecular weight during thermal processing at elevated temperatures. It is also known that both natural and synthetic melanin can be used as thermal stabilizers for polymers [49]. The relationship between melanin dose and the derivative weights was also analyzed. Details on the dose–response of DTG and melanin doses are provided in the Supplementary Figure S4.

#### 2.4.2. Mechanical Properties

**Figure 6:**
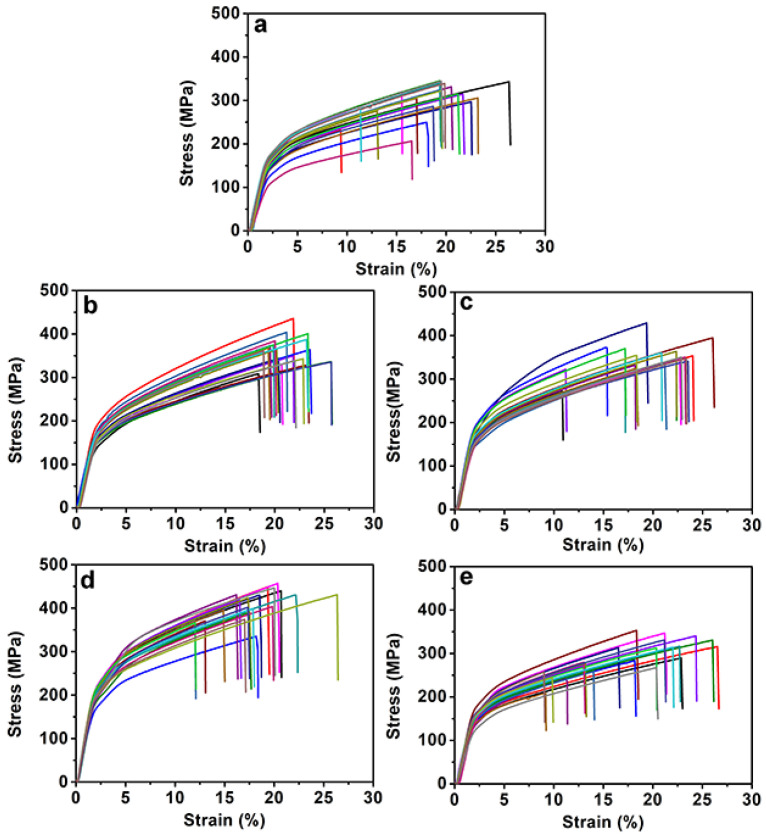
Strain–stress curves of degummed silk fibers of different groups: (**a**) control, (**b**) G1, (**c**) G2, (**d**) G3, and (**e**) G4.

**Figure 7:**
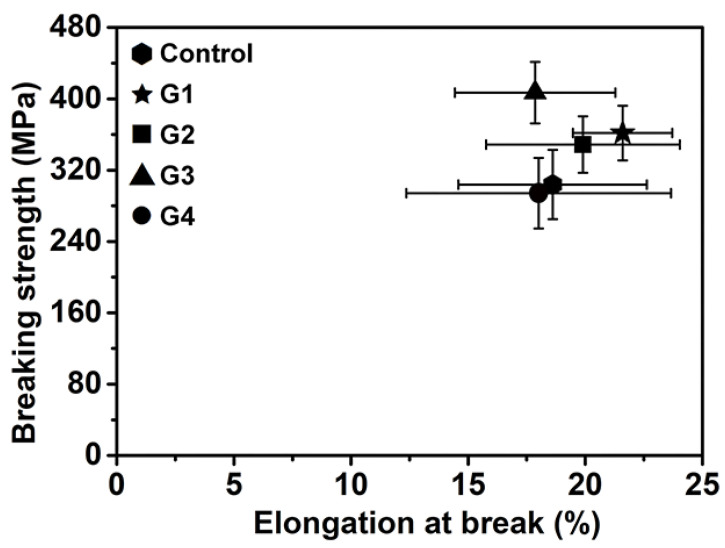
The behavior of the various silks in terms of elongation at break–breaking strength. The error bars represent the standard deviation of breaking strength (ordinate) and elongation at break (abscissa).

Tensile testing was performed to study the effect of melanin on the mechanical properties of silks; the strain–stress plots and elongation at break-breaking strength behavior are shown in Figure 6 and Figure 7 and Supplementary Tables S3–S5. The elongation at break and breaking strength of the silks were found to be closely associated with their secondary structures [50]. Figure 6 depicts the typical stress–strain curves for each treatment, demonstrating the increase in tensile behavior and maximum strain caused by the addition of melanin. It was realized that the addition of melanin had an undeniable influence on the mechanical properties of silk. From Figure 7 and Table 1, when compared to the control, the elongation at break of silks from G1 and G2 was slightly higher, while that of G3 and G4 was comparable. The breaking strength and toughness modulus of the silks produced by G1–G3 were increased to some extent, while they were reduced slightly when from G4, a result that was consistent with the increase in helix and random coil content when compared to the melanin groups. In general, melanin had no significant effect on the mechanical properties of silks, which could be improved to some extent when the concentration of melanin was around 1–3%, as is consistent with the FTIR results. The ability of melanin to improve or degrade the mechanical properties of silk was clearly concentration-dependent. Supplementary Figure S5 depicts the changing behavior of the various groups’ average mechanical properties. However, the precise mechanism is unknown; even so, it is possible that melanin could indeed change the structural composition of silk fibroins from the helix and random coil to β-sheets.

## 3. Conclusions

Melanin, also known as melanin pigment, is a photoprotective natural polymer that has been used in a variety of fields, including nanotechnology, biomedicine, and food processing. Knowing the potential risks of using it as an additive is therefore critical. In this research, the silkworm was used as a model animal to evaluate the biological effects of melanin by feeding silkworm larvae with mulberry leaves sprayed with melanin at different mass concentrations. It is found that melanin is easily cleared from the body by silkworms but still has some negative effects on the growth of silkworm larvae. The histophysiological study showed that the cell morphologies of the silk gland, fat body, and midgut of silkworm were well preserved. The XRD and FTIR results showed that the crystalline and secondary structures of degummed silks were not damaged by melanin. The TGA and tensile test results showed that melanin did not destroy the thermal stability or mechanical properties (breaking strength, elongation at break, and toughness modulus) of the degummed silks; rather, the melanin greatly improved them, thus demonstrating that intrinsically reinforced silks can be simply produced by feeding silkworms with melanin. Overall, this study offers safety evaluation data for melanin, and these data will have guiding importance for the practical use of melanin and an effective way of improving the mechanical performance of silk.

## 4. Materials and Methods

### 4.1. Reagents and Materials

Melanin was purchased from Aladdin reagent company (Shanghai, China). Doubly deionized water was attained from a water purification system (Elix5+Milli-Q, Millipore, Burlington, MA, USA). Shandong Guangtong silkworm egg Group Co., Ltd. (Weifang, China), provided *Bombyx mori* silkworm eggs (strain: Jingsong × Haoyue). 

### 4.2. Characterizations

The histopathological delineation of the silkworm tissues was performed by using a LEICA EZ4HD microscope (Leica, Zürich, Switzerland). Here, after being exposed to melanin, also known as Acid Black 2 (CAS No. 8005-03-6), for 96 h, different tissues (fat body, midgut, and silk gland) of the larvae were taken and stored in formalin before being made into histological sections by Jiangsu University’s affiliated hospital. Approximately 4 silkworms were collected and dissected from each group. During the dissection, the larvae were fixed on foam boards by pins.

An electrothermal thermostat blast drying oven (DHG-9246A, Shanghai Rongfeng Scientific Instrument Co., Ltd., Shanghai, China) was used to dry silkworm cocoons. The diameter and morphology of the degummed silks were obtained by using scanning electron microscopy, SEM (MIRA3, Tescen, Brno, Czech Republic). 

X-ray diffractometer (XRD) (D8 ADNANCE, Bruker, Berlin, Germany) was used to determine the crystalline structure of the degummed silks. 

A Fourier-transform infrared spectroscopy (FTIR) instrument (Nicolet 6700, Thermo Fisher Scientific, Waltham, MA, USA) equipped with a diamond attenuated total reflectance (ATR) accessory was used to investigate the secondary structure of degummed silks. The thermogravimetric analysis (TGA) of degummed silks was characterized by a thermogravimetric analyzer (Mettler Toledo, Greifensee, Switzerland) from 25 to 800 °C (a speed of 10.0 °C min^−1^) in N_2_, at a flow rate of 20.0 mL min^−1^, by Yangzhou University. 

The mechanical properties (breaking strength and elongation at break) of silk fibers were measured by using 20 specimens for each group through a universal tensile-strength tester (Instron 3365, Instron, Norwood, MA, USA), under the condition of a gauge length of 500 mm and an extension of 500 mm min^−1^, in a constant humidity and temperature laboratory (65% RH, 20 °C), by Soochow University’s textile and clothing engineering college. 

### 4.3. Silkworm Rearing and the Intake of Melanin

Silkworms’ (Jingsong × Haoyue) eggs provided by Shandong Guangtong silkworm egg Group Co., Ltd., were incubated in a controlled climatic laboratory until ant silkworms came out (first instar). The temperature was 25 °C, and the relative humidity was 75–80%. At this stage, silkworms were transferred onto bamboo trays and fed with fresh mulberry leaves until maturity. From the 1st day of the 5th instar, a total of 100 silkworms were selected and grouped into 5 experimental sections (20 silkworms in each), among which 1 group was denoted as the control and 4 were denoted as melanin groups. The control group was fed with mulberry leaves sprayed with deionized water; melanin groups were fed with mulberry leaves sprayed with melanin solution at different concentrations (mass concentration: 1%, 2%, 3%, and 4%) until cocooning. Before feeding the larvae, the treated mulberry leaves were allowed to dry at room temperature. According to the concentration of melanin, the melanin groups were named G1, G2, G3, and G4, respectively: G1 = 1%, G2 = 2%, G3 = 3%, and G4 = 4%. Silkworm larvae were fed twice daily, in the morning and at dusk. According to rough estimates, each silkworm larva consumed approximately 2 g of mulberry leaves each time; the mass ratio of mulberry leave to melanin is about 10,000:27 (G1, 1%), 10,000:54 (G2, 2%), 10,000:81(G3, 3%), and 10,000:108 (G4, 4%). The survival rate is the percentage of silkworms that are still alive after 96 h of melanin exposure. Melanin of various weights (1, 2, 3, and 4 g) was mixed with 100 mL distilled water and then ultrasound-treated for 30 min to ensure the even dispersion of the solution.

### 4.4. Silk Reeling

The silkworm cocoons obtained were reaped on the 6th day after spinning. They were then dried in an oven for 4 h at a temperature of 80 °C. They were thereafter kept for a few minutes in boiling water and then transferred into hot water with a temperature of 70 °C. These cocoons were then reeled by using an XJ401 model automatic cocoon-reeling machine (Hangzhou Feiyu technological engineering Co., Ltd., Hangzhou, China). It should be noted that, in the reeling process, 5 cocoons were used together at a time; thereby, the obtained degummed silk fiber comprised 5 single silk fibers. Silks used in this work were reeled in order to get rid of the sericin coated on the surface of the silk fibroin.

### 4.5. Statistical Analysis

The statistical investigation was carried out by using a one-way analysis of variance, followed by an unpaired two-tailed Student’s *t*-test program. A statistical significance *p*-value of less than 0.05 (*p* < 0.05) was considered.

## Figures and Tables

**Table 1:** Mechanical properties of the various silk samples, with average values.

Sample	Elongation at Break (%)	Breaking Strength (MPa)	Toughness Modulus (MPa)
Control	18.61	303.92	42.73
G1	21.59	361.60	56.98
G2	19.90	348.76	51.56
G3	17.87	406.99	55.28
G4	18.01	294.12	40.73

## Data Availability

Not applicable.

## Supplementary Materials

The following supporting information can be downloaded at: https://www.mdpi.com/article/10.3390/toxins14070421/s1, Figure S1: The comparison of (a) average cocoon weight (ACW) and (b) average silk diameter (AD) from different groups. The data for ACW from each group was calculated by the measurement of fifteen cocoons, the one for the AD was calculated by the measurement of ten single silk fibers, the error bars represent the standard deviation of cocoon weight (a) and silk diameter (b), respectively; Figure S2: The deconvolution of FTIR spectra in amide I band of different silk samples. (a) Control, (b) G1, (c) G2, (d) G3 and (e) G4, respectively. The plots exhibit the original spectra (black line), the fitting line (red dotted line), and the deconvoluted traces (three Gaussian curves); Figure S3: Dose-response curve reflecting the relationship between the relative content of β-sheet and the dose of melanin; Figure S4: The dose-response curve between the derivative weights and the melanin dose; Figure S5: The changing behavior of the various groups’ average mechanical properties. (a) elongation at break (%), (b) toughness modulus (MPa), and (c) breaking strength (MPa). The error bars are presented in mean ± SD. ** *p* < 0.001, *** *p* < 0.0005, **** *p* < 0.0001; Table S1: The average length and weight of silkworm, the data for each group was calculated by measuring twenty silkworm larvae; Table S2: The average cocoon weight (ACW) and silk diameter (AD) from different groups. The data for the ACW from each group was calculated by measuring fifteen cocoons, the one for the AD was calculated by measuring ten single silk fibers; Table S3: Elongation at break of different silk samples (%); Table S4: Breaking strength of different silk samples (MPa). Table S5: Toughness modulus of different silk samples (MPa).
