# Supplementary material for: The Evaluation of the Biological Effects of Melanin by Using Silkworm as a Model Animal
Source: Toxins (Basel). 2022 Jun 21;14(7):421. doi: 10.3390/toxins14070421 (PMC9317675; doi:10.3390/toxins14070421)
Supplement: Supplementary file 1 [file toxins-14-00421-s001.zip › toxins-1739557-supplementary.pdf]

toxins

Supplementary Material

The Evaluation of the Biological Effects of Melanin by Using Silkworm as a Model Animal

Vivian Andoh, Liang Chen, Feifei Zhu, Qi Ge, Lin Ma, Qiang Wang and Keping Chen

Results and Discussions

Average length and average weight of silkworms

Table S1. The average length and weight of silkworm, the data for each group was calculated by measuring twenty silkworm larvae.

Sample	Average length (cm)	Average weight (g)
Control (24 h)	4.97	1.764
G1 (24 h)	5.01	1.591
G2 (24 h)	4.79	1.61
G3 (24 h)	4.74	1.704
G4 (24 h)	4.96	1.584
Control (48 h)	5.84	2.633
G1 (48 h)	4.87	2.08
G2 (48 h)	4.79	2
G3 (48 h)	5.09	2.075
G4 (48 h)	5.19	2.014
Control (72 h)	5.8	3.265
G1 (72 h)	5.57	2.717
G2 (72 h)	5.18	2.611
G3 (72 h)	5.4	3.044
G4 (72 h)	5.58	2.773
Control (96 h)	6.2	4.006
G1 (96 h)	5.83	3.566

G2 (96 h)	5.51	3.214
G3 (96 h)	5.94	3.514
G4 (96 h)	5.72	3.346

The comparison of the average cocoon weight (ACW) and average silk diameter (AD) from different groups

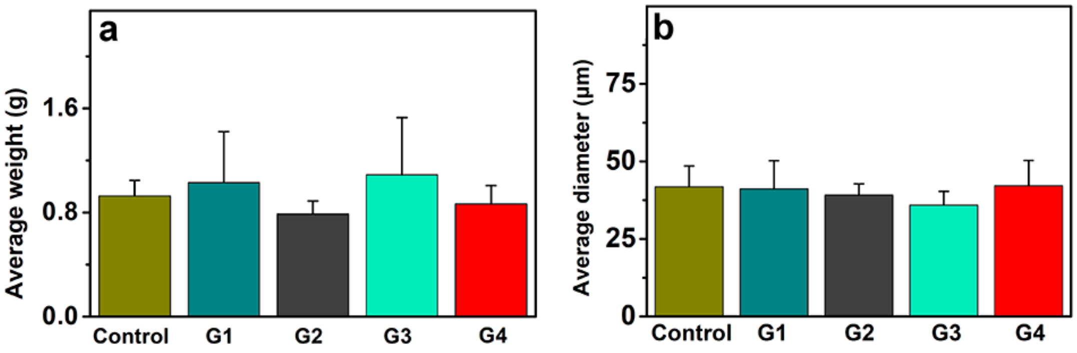

Figure S1. The comparison of (a) average cocoon weight (ACW) and (b) average silk diameter (AD) from different groups. The data for ACW from each group was calculated by the measurement of fifteen cocoons, the one for the AD was calculated by the measurement of ten single silk fibers, the error bars represent the standard deviation of cocoon weight (a) and silk diameter (b), respectively.

Table S2. The average cocoon weight (ACW) and silk diameter (AD) from different groups. The data for the ACW from each group was calculated by measuring fifteen cocoons, the one for the AD was calculated by measuring ten single silk fibers.

Sample	ACW (g)	AD (μm)
Control	0.93	41.84
G1	1.03	41.17
G2	0.79	39.14
G3	1.09	35.91

G4	0.87	42.15

FTIR data

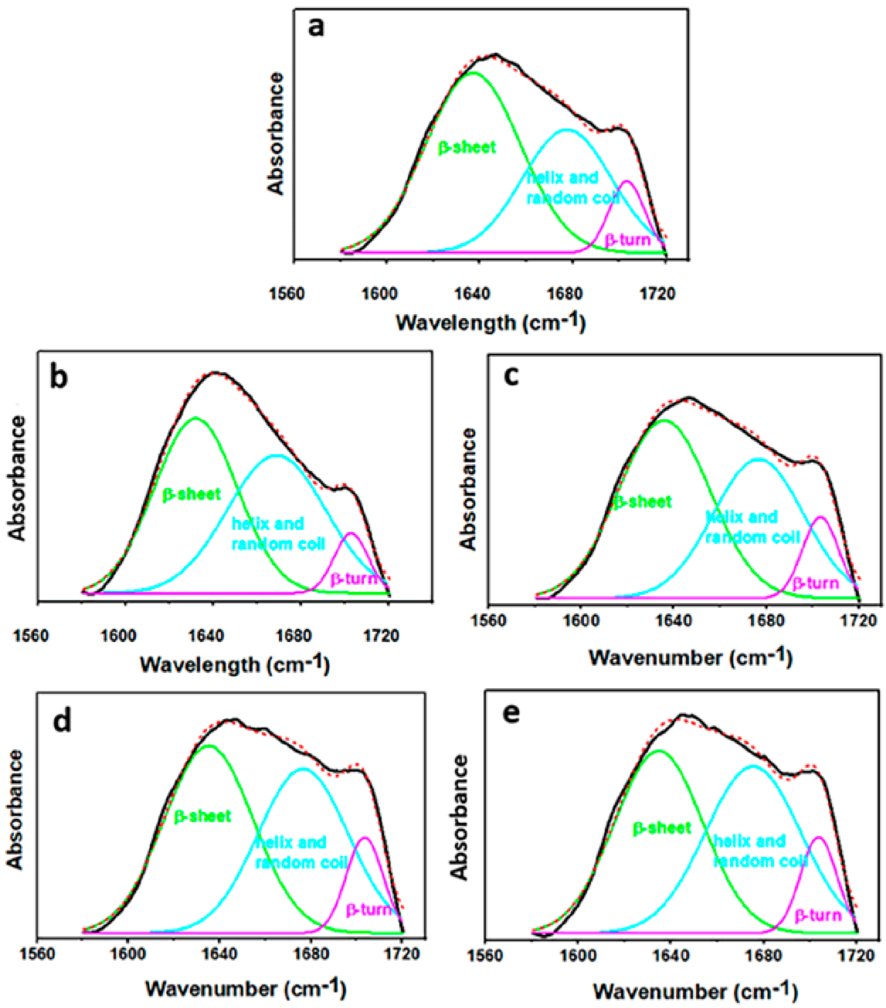

Figure S2. The deconvolution of FTIR spectra in amide I band of different silk samples. (a) Control, (b) G1, (c) G2, (d) G3 and (e) G4, respectively. The plots exhibit the original spectra (black line), the fitting line (red dotted line), and the deconvoluted traces (three Gaussian curves).

A curve reflecting the relationship between the relative content of β-sheet and the dose of melanin is shown in Figure S3. The correlation coefficient is 0.769 (＞0.75), suggesting a linear relationship between relative β-sheet content and melanin dose. An equation is also supplied in the figure, which may help to further evaluate the relative β-sheet contents with the change of the melanin dose.

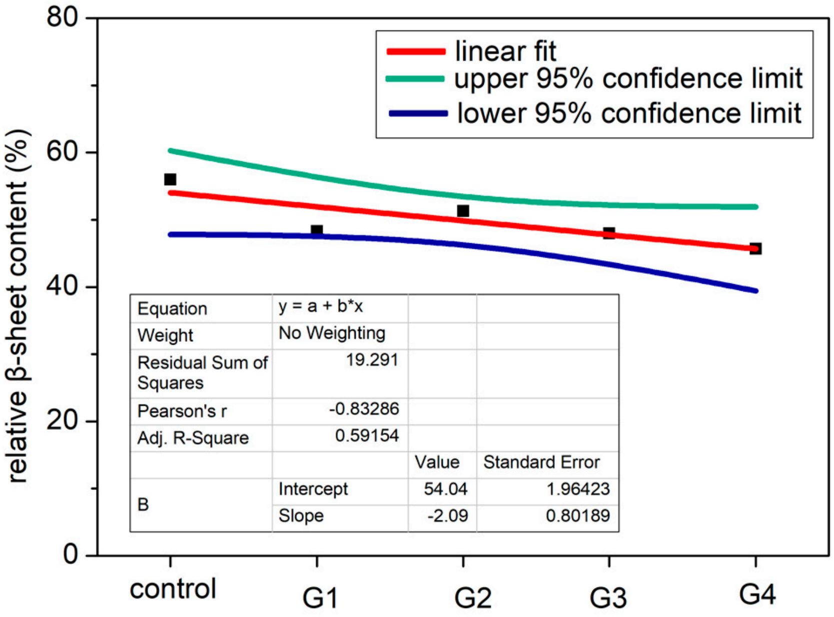

Figure S3. Dose-response curve reflecting the relationship between the relative content of β-sheet and the dose of melanin.

Thermal stability

Derivative thermogravimetric (DTG) data was used to analyze the relationship between the derivative weights and the melanin dose. The result in **Figure S4** shows that with the increase of the melanin dose, the derivative weight decreases gradually. However, the correlation coefficient is 0.616 (＜0.75), suggesting that no efficient linear relation can be established.

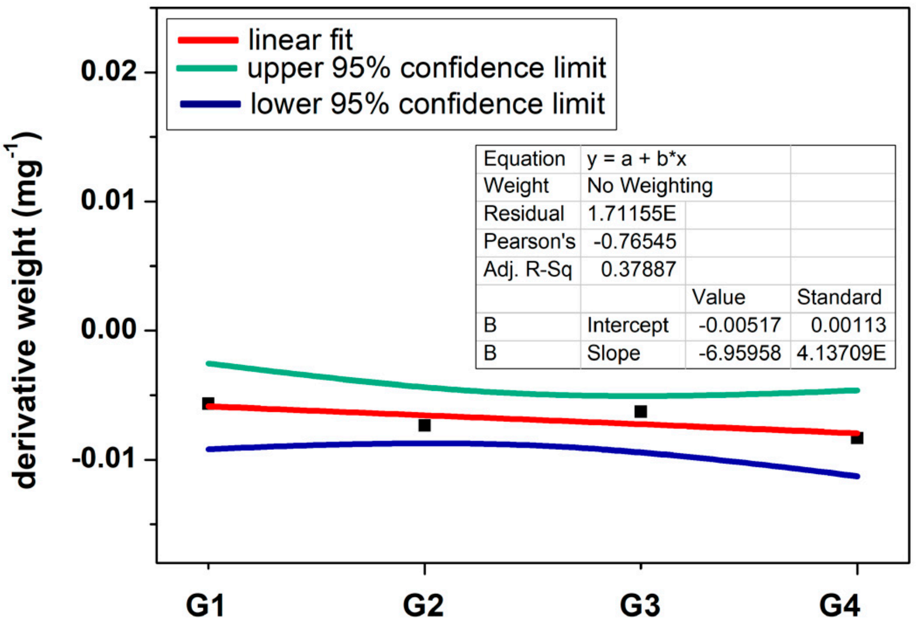

Figure S4. The dose-response curve between the derivative weights and the melanin dose.

Mechanical properties of different silks

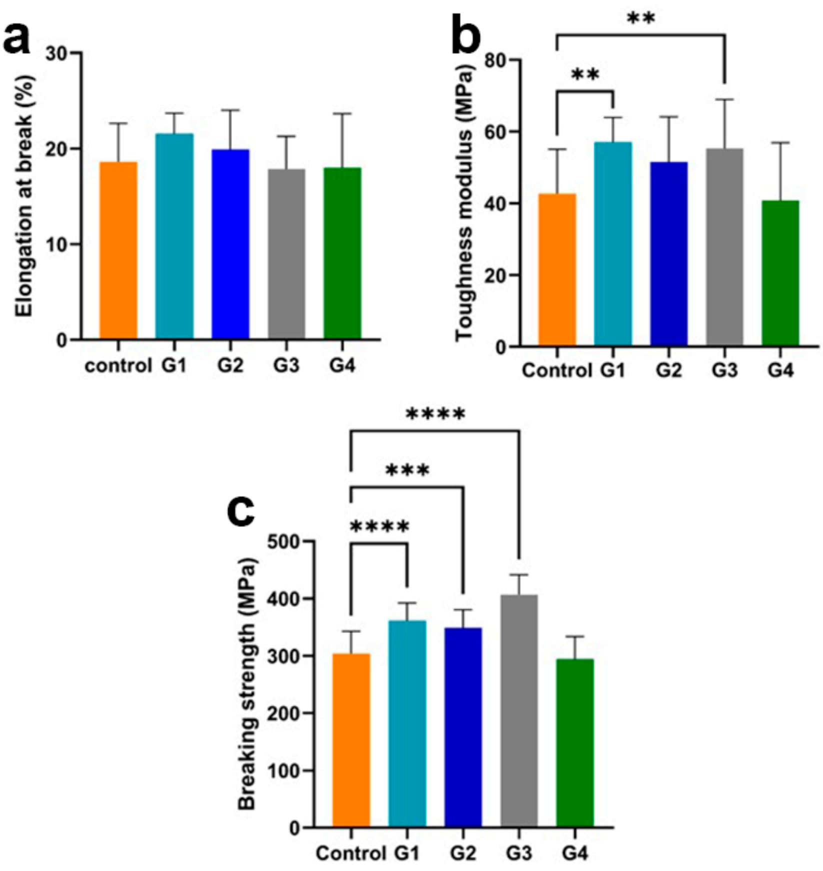

Figure S5. The changing behavior of the various groups' average mechanical properties. (a) elongation at break (%), (b) toughness modulus (MPa), and (c) breaking strength (MPa). The error bars are presented in mean ± SD. ** $p< 0.001$, ***$p<0.0005$, ****$p<0.0001$.

Table S3. Elongation at break of different silk samples (%).

Sample No.	Control	G1	G2	G3	G4
1	26.36	18.36	10.86	20.69	22.86
2	9.36	21.86	24.03	19.36	26.53
3	18.03	23.55	15.19	18.19	18.19
4	15.53	20.70	22.69	20.36	21.19
5	19.36	25.69	22.19	17.52	26.03
6	22.53	20.36	19.36	18.53	16.53
7	21.69	21.86	11.19	16.52	24.36
8	20.53	19.53	18.19	16.19	11.19
9	17.03	23.36	26.03	13.03	18.36
10	13.03	20.19	22.36	17.36	13.19
11	19.53	21.19	21.19	12.03	14.03
12	19.36	19.86	17.03	22.19	22.69
13	23.19	19.36	18.36	14.86	9.19
14	21.19	23.36	17.19	12.03	20.36
15	16.53	20.03	23.19	19.83	13.03
16	11.36	23.19	20.86	17.86	22.03
17	18.69	25.69	23.53	17.36	21.19
18	19.53	22.86	18.36	26.36	9.86
19	19.53	22.03	22.86	20.02	20.36
20	19.86	18.86	23.36	17.03	9.03
average	18.61	21.59	19.90	17.87	18.01

standard deviation	4.02	2.12	4.13	3.43	5.66

Table S4. Breaking strength of different silk samples (MPa).

Sample No.	Control	G1	G2	G3	G4
1	342.98	308.07	271.80	439.57	289.73
2	232.84	435.62	353.27	448.93	315.97
3	249.67	363.85	372.50	334.97	283.83
4	319.69	330.65	344.72	456.99	346.89
5	344.94	336.35	344.18	390.49	330.68
6	296.71	343.36	429.31	429.82	313.29
7	314.72	349.71	322.16	418.48	339.99
8	331.45	373.21	331.81	430.60	241.66
9	305.56	329.37	394.85	370.80	352.98
10	275.62	364.87	363.84	422.96	276.44
11	324.80	404.01	334.13	349.09	258.90
12	339.67	375.07	313.38	429.98	316.66
13	305.78	367.67	324.91	395.78	207.99
14	314.80	400.83	370.40	366.16	312.42
15	206.31	383.86	350.57	403.59	280.21
16	280.85	387.03	360.51	398.54	314.35
17	286.17	335.51	340.95	401.66	330.78
18	322.28	342.96	354.53	430.93	244.80
19	344.34	338.90	350.26	446.35	267.46
20	339.15	361.05	347.08	374.08	257.29
average	303.92	361.60	348.76	406.99	294.12
standard deviation	38.80	30.65	31.72	34.45	39.52

Table S5. Toughness modulus of different silk samples (MPa).

Sample No.	Control	G1	G2	G3	G4
1	67.34	41.16	21.90	68.40	49.51
2	15.69	68.93	62.75	65.19	62.17
3	33.63	62.55	42.44	46.52	38.51
4	36.32	50.14	57.78	69.58	54.75
5	49.72	63.59	56.98	50.89	63.80
6	50.02	51.44	60.93	60.43	38.65
7	50.92	55.18	26.80	52.18	61.59
8	50.26	53.52	44.67	52.57	20.09
9	39.33	57.09	75.26	35.91	48.23
10	27.00	53.60	59.41	55.71	27.66
11	47.02	61.85	51.44	31.24	27.35
12	48.52	54.46	39.75	72.05	53.62
13	52.19	51.85	43.97	44.78	14.55
14	49.69	67.32	47.29	33.26	47.58
15	25.39	55.85	59.15	59.83	26.79
16	24.13	65.78	55.35	54.12	52.08
17	40.04	63.83	59.71	52.33	52.34
18	46.78	57.99	48.56	84.61	18.27
19	50.59	54.24	58.33	68.03	40.21
20	50.05	49.33	58.75	48.05	16.93
average	42.73	56.98	51.56	55.28	40.73
standard deviation	12.40	6.99	12.54	13.67	16.17
